# Editorial Expression of Concern on ‘Protein kinase CK2 phosphorylation regulates the interaction of Kaposi's sarcoma-associated herpesvirus regulatory protein ORF57 with its multifunctional partner hnRNP K’

**DOI:** 10.1093/nar/gkab1294

**Published:** 2021-12-24

In October 2004, NAR published the article ‘Protein kinase CK2 phosphorylation regulates the interaction of Kaposi's sarcoma-associated herpesvirus regulatory protein ORF57 with its multifunctional partner hnRNP K’ by Poonam Malik and J. Barklie Clements (1).

The Editors were alerted in 2021 that the blots depicted in several figures show unusual levels of similarity. As the Authors have not been able to provide the original data, the matter has been referred to the Author's institution for further investigation. In the interim, while the results themselves may not be in question, the Editors advise Readers to examine the details of this study with particular care.

Julian E. Sale, Barry L. Stoddard

Senior Executive Editors

## References

[1] Malik P. , Clements J.B. Protein kinase CK2 phosphorylation regulates the interaction of kaposi's sarcoma-associated herpesvirus regulatory protein ORF57 with its multifunctional partner hnRNP K. Nucleic Acids Res. 2004; 32:5553–5569. 15486205 10.1093/nar/gkh876 PMC524287

